# Chronic Obstructive Pulmonary Disease in the Gulf Cooperation Council: Acute-Care Utilization and Medication Adherence: A PRISMA-ScR Scoping Review

**DOI:** 10.3390/healthcare14131962

**Published:** 2026-07-02

**Authors:** Ahmad Elshafei, Ihab Safi, Adel Aljoaid, Osama Alanazi, Sheffa Almahd

**Affiliations:** 1 Department of Respiratory Therapy, College of Health Sciences, University of Doha for Science and Technology, Doha P.O. Box 24449, Qatar; adel.aljoaid@udst.edu.qa (A.A.); osama.alanazi@udst.edu.qa (O.A.); sheffa.almahd@udst.edu.qa (S.A.); 2 Department of Nursing, College of Health Sciences, University of Doha for Science and Technology, Doha P.O. Box 24449, Qatar; ihab.safi@udst.edu.qa

**Keywords:** chronic obstructive pulmonary disease (COPD), acute exacerbation of COPD (AECOPD), acute-care utilization, hospitalization, readmission, emergency department visits, medication adherence, Gulf Cooperation Council (GCC)

## Abstract

**Background**: Chronic obstructive pulmonary disease (COPD) is a major contributor to morbidity, mortality, and healthcare utilization worldwide. In Gulf Cooperation Council (GCC) countries, the evidence on COPD-related acute-care utilization and medication adherence remains fragmented; hence, this scoping review aimed to map and characterize the available evidence. **Methods**: This scoping review followed the Preferred Reporting Items for Systematic Reviews and Meta-Analyses extension for Scoping Reviews (PRISMA-ScR) checklist. PubMed/MEDLINE, Embase, Scopus, Cochrane Library, and Google Scholar were searched from inception to 22 May 2026. Eligible studies reported COPD-related acute-care utilization and/or medication adherence outcomes in GCC countries. Two reviewers independently screened the studies, extracted the data, and assessed the methodological quality using Joanna Briggs Institute appraisal tools. The findings were synthesized narratively. **Results**: The search identified 328 records, of which 15 studies were included. Most originated from Saudi Arabia, with retrospective cohort and cross-sectional designs predominating. A higher comorbidity burden, obesity, respiratory infection, and disease severity were associated with increased acute-care utilization. Poor medication adherence was associated with increased emergency department visits and hospitalizations. The emerging evidence highlights eosinophilic and obesity-associated COPD phenotypes as potential contributors to clinical outcomes. **Conclusions**: Evidence on acute-care utilization and medication adherence among COPD populations in GCC countries remains limited and heterogeneous. The available studies suggest a healthcare burden, particularly among patients with severe exacerbations and multimorbidity. Future research should prioritize multicenter prospective studies, standardized outcome reporting, and interventions to improve medication adherence and decrease acute-care utilization.

## 1. Introduction

Chronic obstructive pulmonary disease (COPD) remains a major global public health challenge associated with substantial morbidity, mortality, impaired quality of life, and increasing healthcare resource utilization across developed and developing regions [1,2]. Acute exacerbations of COPD (AECOPDs) are among the principal drivers of disease burden and frequently result in emergency department (ED) visits, hospitalization, intensive care unit (ICU) admission, prolonged hospital length of stay (LOS), and recurrent readmissions [1]. According to the World Health Organization (WHO), COPD was responsible for an estimated 3.5 million deaths worldwide in 2021, accounting for approximately 5% of all deaths [3].

Severe exacerbations often require hospitalization and are associated with accelerated lung-function decline, poorer health-related quality of life, and increased mortality risk, particularly among patients with multimorbidity, obesity, respiratory infections, and delayed outpatient management [4,5]. Adherence to prescribed pharmacologic therapy is an important component of COPD management, as appropriate maintenance treatment can reduce the symptoms, improve the health status, decrease the exacerbation frequency, and lower the risk of hospitalization [6].

Although the burden of COPD is globally recognized, understanding how exacerbations translate into acute-care utilization and healthcare-system demands requires region-specific evidence, particularly in settings with unique demographic, environmental, and healthcare characteristics.

In Gulf Cooperation Council (GCC) countries, including Saudi Arabia, the United Arab Emirates (UAE), Oman, Kuwait, Qatar, and Bahrain, COPD-related healthcare utilization is expected to increase due to population aging, persistent tobacco exposure, environmental and occupational pollutants, obesity, and rising chronic disease prevalence. Despite the growing clinical and economic importance of COPD in the region, the evidence regarding the hospitalization outcomes, ICU utilization, readmissions, mortality, ED utilization, and healthcare resource consumption remains fragmented and geographically uneven. Most published studies originate from Saudi Arabia and the UAE, whereas evidence from Qatar, Bahrain, Oman, and Kuwait remains comparatively limited [4,7,8]. This heterogeneity and relative scarcity of regional evidence complicate efforts to characterize the overall burden of COPD-related acute-care utilization across GCC healthcare systems.

Medication adherence represents a potentially modifiable determinant of COPD outcomes that may influence the exacerbation frequency, ED utilization, and hospitalization outcomes. Previous regional studies, including the ADCARE study, demonstrated suboptimal adherence to COPD treatments among patients in Saudi Arabia and neighboring countries and suggested associations between lower adherence and poorer clinical outcomes [9]. Additional studies from GCC settings have reported associations between reduced medication adherence and increased ED visits, poorer symptom control, and lower health-related quality of life [10,11]. However, adherence measures and outcome definitions vary substantially across studies, limiting comparability and synthesis.

Given the heterogeneity, the limited number of studies, and the variability in methodologies and outcome definitions across the available GCC literature, a scoping review methodology was considered more appropriate than a systematic review. Therefore, the aim of this review was to map and characterize the available evidence regarding COPD related acute-care utilization and medication adherence across GCC countries.

## 2. Methods

This scoping review was conducted in accordance with the Preferred Reporting Items for Systematic Reviews and Meta-Analyses extension for Scoping Reviews (PRISMA-ScR) guidelines [12], and the completed PRISMA-ScR checklist is provided in the Supplementary Materials. A review protocol was developed before study screening commenced; public registration on Open Science Framework (OSF) Registries occurred after completion of the review process on 29 April 2026, and due to the expanded search, it was further updated on 3 June 2026, and is, therefore, reported transparently as a post hoc registration (DOI: 10.17605/OSF.IO/DSQBG).

Eligibility criteria were established a priori using the Population–Concept–Context framework recommended for scoping reviews. The population included adult patients diagnosed with COPD. The concepts of interest were COPD-related acute-care utilization and medication adherence outcomes across GCC countries. Acute-care utilization outcomes included hospitalization, intensive care unit (ICU) admission, emergency department (ED) visits, mortality, readmission outcomes, and length of stay. Because hospitalization is generally considered a marker of the severe acute exacerbation of COPD (AECOPD), studies evaluating hospitalized COPD populations and severe exacerbation cohorts were of particular interest. Where studies reported exacerbation severity classifications, the original study definitions were retained. Medication adherence outcomes included adherence or treatment utilization measures and tools as reported by the studies. The context was limited to Gulf Cooperation Council (GCC) countries, including Saudi Arabia, the United Arab Emirates (UAE), Oman, Kuwait, Qatar, and Bahrain. Studies were eligible if they reported GCC-specific data and used observational or interventional designs. Studies were excluded if they were not COPD-focused; lacked extractable GCC-specific data; included mixed respiratory populations without separable COPD-specific outcomes; focused exclusively on physiological outcomes without utilization or adherence endpoints; represented reviews, editorials, commentaries, protocols, conference abstracts without extractable data; or were other non-primary research publications. The full inclusion and exclusion criteria are provided in the Supplementary Materials.

Searches were conducted in PubMed/MEDLINE, Embase, Scopus, Cochrane Library, and Google Scholar from database inception to 22 May 2026. Database-specific search strings, search limits, and retrieval counts are provided in the Supplementary Materials. Google Scholar was searched as a supplementary source to identify additional potentially relevant studies and regional literature not indexed in major biomedical databases.

Records identified through database searching were exported into RefWorks reference-management software (Ex Libris, 2026) for duplicate removal and screening [13]. Two reviewers independently screened titles and abstracts, followed by the full-text review of potentially eligible studies. Disagreements were resolved through discussion and consensus. Full-text exclusion reasons are reported explicitly within the PRISMA flow diagram and Supplementary Materials.

A standardized data-charting form was used for data extraction; the form was piloted on a subset of included studies and refined iteratively to ensure consistency and completeness. Two reviewers independently performed data charting and methodological assessment. The extracted variables are outlined in the data extraction form in the Supplementary Materials. Data management and collection were conducted using Microsoft Excel.

Consistent with scoping-review methodology, studies were not excluded based on their methodological quality. Nevertheless, the methodological rigor was assessed to improve the interpretation of the findings. Critical appraisal was conducted using the Joanna Briggs Institute (JBI) critical appraisal tools appropriate for each study design [14]. The JBI Cohort Checklist was applied to retrospective and prospective cohort studies, while the JBI Analytical Cross-Sectional Checklist was used for cross-sectional studies. Ecological and time-series studies were evaluated using an adapted JBI quasi-experimental framework appropriate to the respective methodology. Two reviewers independently completed the methodological appraisal, with disagreements resolved through discussion and consensus. Studies were assessed across multiple methodological domains, including participant selection, validity of exposure and outcome measurement, confounding management, and completeness of outcome reporting. Within the appraisal tables, “Yes” indicates that the methodological criterion was adequately addressed (lower risk of bias), “No” indicates that the criterion was not adequately addressed (higher risk of bias), and “Unclear” indicates insufficient reporting to determine methodological adequacy. Overall appraisal ratings were derived through reviewer consensus based on the number and significance of identified methodological limitations.

The findings were synthesized narratively according to major thematic domains, including hospitalization outcomes, healthcare resource utilization, medication adherence, and emerging COPD phenotypes. This approach was selected because the available evidence base was limited, heterogeneous, and predominantly observational in nature, supporting the appropriateness of a scoping-review methodology rather than a formal systematic. Furthermore, the interpretation of associations between medication adherence and healthcare utilization outcomes was intentionally cautious throughout the synthesis, particularly for retrospective and cross-sectional studies where confounding and causal inference limitations were substantial.

## 3. Results

### 3.1. Study Selection

**Figure 1:**
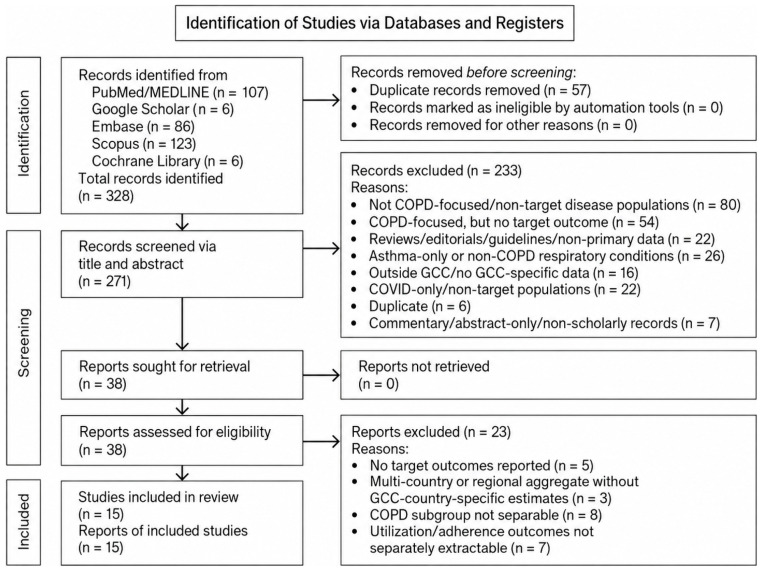
Flowchart of the article selection process.

A total of 328 records were identified through searching PubMed/MEDLINE (n = 107), Embase (n = 86), Scopus (n = 123), Google Scholar (n = 6), and Cochrane Library (n = 6). Following duplicate removal (n = 57), 271 records underwent title and abstract screening. Of these, 233 records were excluded. Thirty-eight reports underwent full-text eligibility assessment, with 23 reports excluded for reasons including non-extractable GCC-specific data and the absence of acute-care utilization or adherence outcomes. Ultimately, 15 studies were included in the final synthesis. The PRISMA-ScR flow diagram of the study selection process is provided in Figure 1.

### 3.2. Study Characteristics

**Figure 2:**
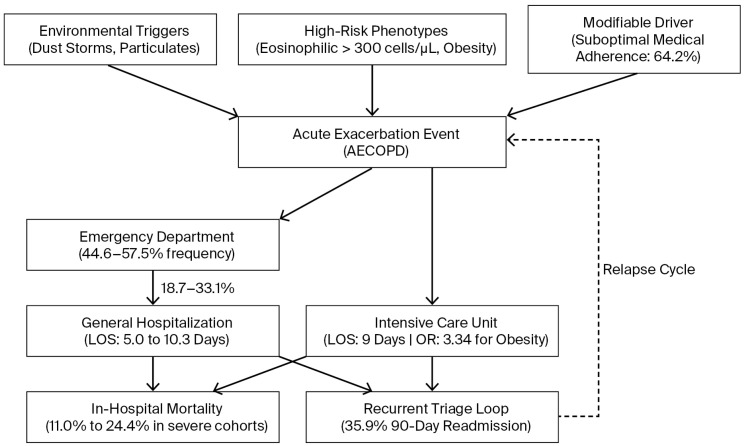
Conceptual pathway of COPD burden-related domains. Caption: Conceptual framework illustrating the pathways linking environmental exposures, high-risk COPD phenotypes, medication adherence, acute exacerbations, and subsequent acute-care utilization and clinical outcomes in GCC COPD populations. The framework summarizes the relationships identified across the included studies, including emergency department utilization, hospitalization, intensive care unit (ICU) admission, mortality, and readmission.

**Figure 3:**
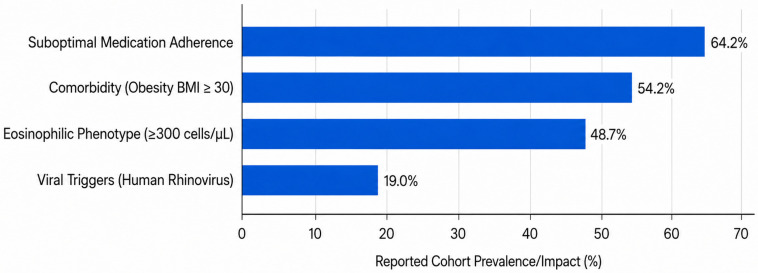
Descriptive prevalence estimates of selected factors reported among COPD populations in the included GCC studies. Caption: Values are derived from individual studies and presented for descriptive purposes only. They should not be interpreted as pooled estimates, directly comparable frequencies, or measures of the relative contribution of these factors to COPD admission or readmission.

The included literature addressed multiple domains related to COPD burden, including hospitalization outcomes, ICU utilization, mortality, LOS, medication adherence, ED visits, eosinophilic COPD phenotypes, obesity-associated outcomes, and prognostic indicators during AECOPDs, as illustrated in Table 1 and Figure 2. The frequency of the reported reasons for COPD admission and readmission across the included GCC sources is shown in Figure 3.

Several studies specifically focused on acute-care utilization outcomes among hospitalized COPD populations. Alaithan et al. [4] evaluated hospital and intensive care unit (ICU) outcomes among patients admitted with acute exacerbations of COPD in Saudi Arabia, while Onadeko et al. [7] explored prognostic factors during COPD exacerbations in Kuwait. Additional studies assessed the LOS and comorbidity burden in tertiary-care populations, ICU admission predictors, and disease severity scoring systems, such as the DECAF score [16,17].

Medication adherence and healthcare utilization represented another major thematic area. Studies from Saudi Arabia and multinational Middle Eastern cohorts examined associations between medication adherence, ED utilization, and healthcare resource consumption [16,21].

More recent studies have expanded the literature to include eosinophilic COPD phenotypes, obesity-associated outcomes, and respiratory symptom associations with clinical outcomes [16,17,19,21], suggesting a growing shift toward multidimensional COPD management frameworks within GCC healthcare systems.

### 3.3. Critical Appraisal Summary

Based on the JBI critical appraisal, the evidence base was predominantly moderate quality, with two studies rated as high quality, two studies rated as low-to-moderate quality, and the remainder rated as moderate quality (Table 2). Common methodological limitations included inadequate control of confounding variables, unclear reporting of key population characteristics, and potential measurement bias in studies relying on self-reported adherence data. Several retrospective cohort and cross-sectional studies did not fully address confounding, which reduced the confidence in causal interpretations. In contrast, multicenter and validation cohort studies generally demonstrated stronger methodological rigor. Overall, the available evidence provides a preliminary overview of COPD related acute-care utilization and medication adherence in GCC countries; however, interpretation should remain cautious because most included studies were observational, several did not adequately control confounding, and the methodological heterogeneity was substantial.

### 3.4. Acute-Care Utilization Outcomes

#### 3.4.1. Hospitalization and Length of Stay

Hospitalization-related outcomes were frequently reported across the included studies (Table 1). The LOS varied substantially depending on the disease severity, ICU admission status, and comorbidity burden. Alaithan et al. [4] reported a median ICU LOS of nine days among critically ill COPD patients admitted to ICU settings in Saudi Arabia. Similarly, Alotaibi et al. [17] reported a median hospital LOS of five days in a tertiary-care Saudi Arabian population and identified that male sex was associated with an additional 1.30 days of hospitalization, while cancer/malignancy was associated with an additional 2.73 days. Increased comorbidity burden, obesity, respiratory infections, and advanced disease severity were associated with prolonged hospitalization durations [7,17]. Alotaibi et al. [17] identified significant associations between COPD-related comorbidities and increased LOS in tertiary-care populations. Al-Jahdali et al. [15] also reported prolonged LOS among patients admitted with pulmonary diseases, including COPD cohorts, particularly among patients with complex comorbidity profiles. In Kuwait, Onadeko et al. [7] reported a mean hospital LOS of 10.3 days and highlighted the prognostic significance of exacerbation severity in determining hospitalization outcomes and disease progression during acute exacerbations.

#### 3.4.2. Intensive Care Unit Admission and Mortality

Several studies evaluated ICU utilization and mortality outcomes among COPD populations (Table 1 and Table 3). Alaithan et al. [4] demonstrated that severe COPD exacerbations requiring ICU admission were associated with increased morbidity and mortality, reporting an ICU mortality rate of 6% and a hospital mortality rate of 11% among ICU-managed exacerbation patients in Saudi Arabia. Obesity-related studies conducted in Saudi Arabia also identified significant associations between obesity, ICU admission, and comorbidity burden among COPD patients [5]. Almarshoodi et al. [16] validated the DECAF score within GCC populations and demonstrated its usefulness in predicting the disease severity and hospital mortality among patients hospitalized with AECOPD. Moreover, in that study mortality among hospitalized exacerbation patients was 24.4%, with a 90-day readmission rate of 35.9% [16].

#### 3.4.3. Emergency Department Utilization and Readmissions

Alshehri and Alshibani [10] reported that 44.6–57.5% of COPD patients in Saudi Arabia utilized ED services within one year, with 18.7–33.1% requiring hospitalization following ED presentation. Lower medication adherence was significantly associated with increased ED visits among COPD patients in Saudi Arabia. The BREATHE study [8] further demonstrated high healthcare resource utilization among COPD populations across Middle Eastern and North African regions, including GCC countries. Regarding readmissions and recurrent utilization, the multicenter UAE cohort study by Almarshoodi et al. [16] reported a high 90-day recurrent hospital readmission rate of 35.9% (184/512) among patients with confirmed or physician-diagnosed COPD. Additionally, Al Sibani et al. [19] found that patients with an eosinophilic AECOPD phenotype (>0.3 × 10^9^ cells/L) experienced significantly more recurrent hospitalizations, with a median of one annual readmission compared with zero for non-eosinophilic patients (Table 1 and Table 3).

### 3.5. Medication Adherence and Related Outcomes

Medication adherence was the focus of Kokturk et al. [9] Through the ADCARE study, it was demonstrated that the prevalence of low medication adherence was 64.2% among COPD patients in Saudi Arabia and neighboring regions, and a significant predictor of low adherence included living in Saudi Arabia rather than Turkey (OR = 3.20). Alshehri and Alshibani [10] reported that lower medication adherence was significantly associated with increased ED visits among COPD patients in Saudi Arabia. More recent evidence by Saja et al. [11] demonstrated that improved medication adherence was associated with better HRQoL outcomes among COPD patients. Collectively, adherence-related findings suggest that medication adherence remains a persistent challenge within GCC COPD populations and may represent an important target for future disease-management interventions (Table 1 and Table 3).

### 3.6. Emerging Clinical Phenotypes and Predictor Factors

Alshehri et al. [21] reported a 48.7%prevalence of eosinophilic COPD in Saudi Arabia, while Al Sibani et al. [19] demonstrated that 42.2% of AECOPD patients in Oman had elevated peripheral eosinophil counts, which were associated with clinically significant disease characteristics and a higher readmission risk. The respiratory symptom burden was also identified as an important determinant of clinical outcomes. Alqarni et al. [18] reported significant associations between the respiratory symptom severity and poorer COPD-related outcomes.

Additional evidence from Abu Dhabi demonstrated the potential role of human rhinovirus in COPD exacerbations, highlighting the contribution of viral respiratory infections to the exacerbation burden and acute-care utilization [20]. Furthermore, Bener et al. [22] reported increased COPD admissions in Qatar during dust events, underscoring the role of environmental exposures in acute-care utilization (Table 1 and Table 3).

## 4. Discussion

This scoping review synthesized the available evidence regarding acute-care utilization and medication adherence among COPD populations in GCC countries, which demonstrated that COPD is associated with a substantial burden on healthcare systems across the region through frequent hospitalizations, prolonged LOS, ICU admissions, recurrent ED utilization, and elevated healthcare resource consumption [4,5,7].

Most of the included studies originated from Saudi Arabia, reflecting a relative concentration of COPD research activity. In contrast, evidence from other GCC countries such as Qatar, Bahrain, Oman, and Kuwait remained comparatively limited, highlighting important regional research gaps. This imbalance underscores the need for broader multicenter and multinational investigations to improve the understanding of COPD burden across diverse GCC healthcare systems.

Hospitalization and LOS outcomes emerged as major themes within the included literature. Multiple studies identified associations between prolonged hospitalization and increased disease severity, obesity, comorbidity burden, and respiratory infections [5,17]. A comparison with other studies conducted around the world reinforces the importance of these results. The rates of readmission for COPD highly differ between countries, depending on the healthcare system, coding, severity criteria, and follow-up definitions. In other studies, the rate of 30-day readmissions was between 8.8% and 26.0%, whereas the rate of 90-day readmissions was between 17.5% and 39.0% [23]. Considering this information, the 90-day readmission rate estimated for the UAE (35.9%) falls in the upper range of the published readmission rates [16,23]. These findings are consistent with the international COPD literature demonstrating that multimorbidity and greater disease severity are associated with an increased risk of hospital readmission following COPD exacerbation [23]. 

ICU utilization and mortality outcomes also represented important areas of investigation. Studies evaluating critically ill COPD populations reported substantial morbidity and mortality among patients requiring ICU-level care [4]. The validation of the DECAF score within GCC populations represents an important contribution to regional COPD management, as prognostic scoring systems may assist clinicians in identifying high-risk patients and optimizing resource allocation during acute exacerbations [16].

Medication adherence emerged as another key factor associated with COPD outcomes within the region. Several studies consistently demonstrated associations between poor adherence and increased ED utilization, worse HRQoL, and increased healthcare resource consumption [9,10,11]. These findings suggest that structured adherence-support strategies, patient education, pulmonary rehabilitation, and long-term disease-management programs may be beneficial within GCC healthcare systems.

Recent studies additionally emphasized the growing recognition of COPD heterogeneity within the GCC population. Eosinophilic phenotypes, obesity-associated COPD, psychological symptom burden, and viral-associated exacerbations were increasingly recognized as clinically important contributors that may be associated with disease progression and acute-care utilization [19,20,21]. These findings suggest a potential movement toward personalized and phenotype-driven COPD management approaches in the region.

Overall, the findings of this review suggest that COPD-related acute-care utilization and medication adherence are major clinical and public-health concerns across GCC countries and highlight the need for coordinated multicenter research, standardized outcome reporting, and strengthened continuity-of-care strategies to improve COPD management across the region.

### Strengths and Limitations

This review has several strengths. It provides syntheses regarding COPD acute-care utilization and medication adherence across GCC countries, incorporating evidence from multiple databases and including studies evaluating diverse acute-care utilization and medication adherence outcomes, allowing for a broader characterization of COPD burden across the region.

However, several limitations should be acknowledged. First, substantial geographic disparities in the available evidence were identified, with most studies originating from Saudi Arabia and the UAE, with relatively limited evidence from Bahrain, Qatar, Kuwait, and Oman. Second, the methodological heterogeneity between the studies was considerable, particularly regarding exacerbation definitions and outcome reporting. Some studies defined exacerbations using hospital admissions or ED visits, whereas others relied on physician diagnosis or coded administrative outcomes.

Third, many of the included studies were retrospective and single center in design, limiting the generalizability and causal inference. Confidence intervals, adjusted effect estimates, and standardized outcome measures were inconsistently reported across studies, limiting deeper quantitative interpretation. Fourth, the review was restricted to English-language publications, which may have resulted in the omission of relevant evidence published in Arabic or that reported in local regional sources. Finally, the observational nature of the included studies prevents the establishment of causal relationships between medication adherence, exacerbation risk, and hospitalization outcomes.

## 5. Conclusions

Evidence regarding COPD-related acute-care utilization and medication adherence across GCC countries remains limited, geographically uneven, and methodologically heterogeneous. The existing studies show there are substantial burdens related to hospitalization, ICU admission, readmissions, mortality, prolonged LOS, and medication non-adherence among patients with COPD in several GCC healthcare systems. However, the current evidence base remains insufficient for robust regional benchmarking or definitive causal inference.

Future prospective studies are needed to improve the evidence quality, strengthen the continuity of care, and support healthcare planning across GCC countries. Enhanced adherence interventions, transitional-care programs, and preventive strategies targeting infection-related exacerbations may help reduce avoidable acute-care utilization and improve long-term COPD outcomes throughout the region.

## Figures and Tables

**Table 1:** Characteristics and key findings of studies reporting acute-care utilization and medication adherence outcomes among patients with COPD in GCC countries.

Study	Country	Study Design	Sample Size	COPD Definition	Outcome	Value
Alaithan et al. [4]	Saudi Arabia	Retrospective cohort	119	GOLD-defined COPD (spirometry-confirmed)	Hospital mortality in ICU-managed exacerbation patients	11% (13/119 patients)
Al-Jahdali et al. [15]	Saudi Arabia	Retrospective cohort	1358	Not explicitly reported	Obesity (BMI ≥ 30) prevalence in COPD subgroup and total pulmonary cohort	54.2% in COPD subgroup; 42.5% in total cohort
Almarshoodi et al. [16]	UAE	Multicenter cohort	512	Confirmed/physician-diagnosed COPD	90-day recurrent readmission rate following hospitalization	35.9% (184/512)
Almarshoodi et al. [16]	UAE	Multicenter cohort	512	Confirmed/physician-diagnosed COPD	Inpatient mortality among hospitalized severe exacerbation patients	24.4% (125/512)
Almarshoodi et al. [16]	UAE	Multicenter cohort	512	Confirmed/physician-diagnosed COPD	DECAF score validation for inpatient mortality	AUROC 0.80 (95% CI: 0.80–0.87)
Almarshoodi et al. [16]	UAE	Multicenter cohort	512	Confirmed/physician-diagnosed COPD	Mean hospital LOS and DECAF stratification ranges	Overall mean 14.3 (32.5) days; score 0: 3.6 ± 2.0 days; Score 6: 29.8 ± 31.4 days
Alotaibi et al. [17]	Saudi Arabia	Cross-sectional	629	Confirmed/physician-diagnosed COPD	Median hospital LOS	5 days (IQR 3–9)
Alotaibi et al. [17]	Saudi Arabia	Cross-sectional	629	Confirmed/physician-diagnosed COPD	High baseline comorbidity burden in hospitalized COPD cohorts	Hypertension 78.8%; pneumonia; 67.8%
Alqarni et al. [5]	Saudi Arabia	Retrospective cohort	474	Confirmed/physician-diagnosed COPD	ICU admission rate	Obese patients; 16%, overweight patients; 12%
Alqarni et al. [18]	Saudi Arabia	Cross-sectional	214	Confirmed/physician-diagnosed COPD	Hospital admission within previous year among COPD cohort	42.0% (91/214)
Al Sibani et al. [19]	Oman	Retrospective cohort	102	GOLD-defined COPD (spirometry-confirmed)	Eosinophilic AECOPD phenotype prevalence (>0.3 × 10^9^ cells/L)	42.2%
Al Sibani et al. [19]	Oman	Retrospective cohort	102	GOLD-defined COPD (spirometry-confirmed)	Hospital LOS by eosinophilic phenotype	Total: 4 (3–7) days; normal eosinophils: 5 (4–7) days; high eosinophils: 4 (3–6) days (*p* = 0.02)
Alsayed et al. [20]	UAE	Retrospective cohort	58	Confirmed/physician-diagnosed COPD	Human rhinovirus (HRV) positivity during acute exacerbation	19% (11/58 patients)
Alshehri and Alshibani [10]	Saudi Arabia	Retrospective cohort	266	Confirmed/physician-diagnosed COPD	ED utilization within 1 year among COPD cohorts	High adherence: 44.6% vs. low adherence: 57.5% (*p* < 0.036)
Alshehri and Alshibani [10]	Saudi Arabia	Retrospective cohort	266	Confirmed/physician-diagnosed COPD	Hospitalization following ED presentation	High adherence: 18.7% vs. low adherence: 33.1% (*p* = 0.007)
Alshehri et al. [21]	Saudi Arabia	Retrospective cohort	156	GOLD-defined COPD (spirometry-confirmed)	Eosinophilic COPD phenotype prevalence (≥300 cells/µL)	48.7% (76/156 patients)
Bener et al. [22]	Qatar	Prospective population-based ecological/time-series	N/A (population-level time-series analysis)	Not explicitly reported	Hospital admissions associated with air pollution	Average 3.4 admissions/day
Kokturk et al. [9]	Saudi Arabia/Turkey	Cross-sectional	405	Confirmed/physician-diagnosed COPD	* Low medication adherence prevalence (MMAS-8 < 6)	64.2%
Kokturk et al. [9]	Saudi Arabia/Turkey	Cross-sectional	405	Confirmed/physician-diagnosed COPD	Association between country and medication adherence	Saudi Arabia associated with lower medication adherence than Turkey (OR 3.20, *p* = 0.0001)
Onadeko et al. [7]	Kuwait	Observational cohort	74	Confirmed/physician-diagnosed COPD	Standard hospitalization duration profile for acute management	Majority LOS: 7–14 days
Onadeko et al. [7]	Kuwait	Observational cohort	74	Confirmed/physician-diagnosed COPD	Acute inpatient mortality	19% mortality (14/74 patients)
Polatli et al. [8]	MENA Region	Multinational observational	1392	Not explicitly reported	Mean nights hospitalized	Saudi Arabia: 4.66 ± 7.91 nights; UAE: 3.8 ± 5.03 nights
Saja et al. [11]	Saudi Arabia	Cross-sectional	424	GOLD-defined COPD (spirometry-confirmed)	* Mean MARS-5 score and high adherence prevalence (≥23)	Mean 21.57 ± 4.34; 56.4% high adherence

This table summarizes characteristics and key findings of studies. The reported values are presented as a percentage, mean ± standard deviation (SD), median with interquartile range (IQR), or effect estimate with a 95% confidence interval (CI), as provided in the original studies. * Subjective self-reporting of medication adherence by patient.

**Table 2:** Joanna Briggs Institute (JBI) critical appraisal.

Study	Study Design	JBI Checklist Used	Selection Bias	Measurement Validity	Confounding Addressed	Outcome Reporting	Overall Appraisal
Alaithan et al. [4]	Retrospective cohort	JBI Cohort Checklist	Yes	Yes	No	Yes	Moderate
Al-Jahdali et al. [15]	Retrospective cohort	JBI Cohort Checklist	Yes	Yes	No	Yes	Moderate
Almarshoodi et al. [16]	Multicenter cohort	JBI Cohort Checklist	Yes	Yes	Yes	Yes	High
Alotaibi et al. [17]	Cross-sectional	JBI Analytical Cross-Sectional Checklist	Yes	Yes	No	Yes	Moderate
Alqarni et al. [5]	Retrospective cohort	JBI Cohort Checklist	Yes	Yes	Yes	Yes	High
Alqarni et al. [18]	Cross-sectional	JBI Analytical Checklist	Yes	Yes	Unclear	Yes	Moderate
Alsayed et al. [20]	Retrospective cohort	JBI Cohort Checklist	Yes	Yes	Unclear	Yes	Moderate
Alshehri et al. [21]	Retrospective cohort	JBI Cohort Checklist	Yes	Yes	Unclear	Yes	Moderate
Alshehri and Alshibani [10]	Retrospective cohort	JBI Cohort Checklist	Yes	Yes	Unclear	Yes	Moderate
Al Sibani et al. [19]	Retrospective cohort	JBI Cohort Checklist	Yes	Unclear	No	Yes	Moderate
Bener et al. [22]	Ecological/time-series	Adapted JBI Quasi-Experimental	Yes	Yes	No	Yes	Moderate
Kokturk et al. [9]	Cross-sectional	JBI Analytical Cross-Sectional Checklist	Yes	No (self-report bias)	No	Yes	Low–Moderate
Onadeko et al. [7]	Observational cohort	JBI Cohort Checklist	Yes	Unclear	No	Yes	Moderate
Polatli et al. [8]	Multinational observational	JBI Analytical Cross-Sectional Checklist	Yes	Yes	Unclear	Yes	Moderate
Saja et al. [11]	Cross-sectional	JBI Analytical Cross-Sectional Checklist	Yes	No (self-report bias)	No	Yes	Low–Moderate

Methodological characteristics and quality appraisal of studies included in the scoping review. This table summarizes the study design, JBI critical appraisal tool used, assessment domains, and overall methodological quality ratings.

**Table 3:** Health-related outcomes and predictor factors (where reported).

Study	Outcome	Predictor	Effect Estimate
Alaithan et al. [4]	In-hospital mortality	Glasgow Coma Scale score	OR 1.24 (95% CI: 1.04–1.76)
Alaithan et al. [4]	In-hospital mortality	Current smoking	OR 0.062 (95% CI: 0.010–0.389)
Al-Jahdali et al. [15]	Length of stay	Age, sex, obesity, and comorbidities	Not reported
Almarshoodi et al. [16]	Inpatient mortality prediction	DECAF score	AUROC 0.80 (95% CI: 0.80–0.87)
Almarshoodi et al. [16]	Disease severity and prolonged LOS	Higher DECAF scores (Score 6)	29.8 ± 31.4 days LOS (*p* = 0.008)
Alotaibi et al. [17]	Increased hospital LOS	Male sex	+1.30 days (95% CI: 0.15–2.77)
Alotaibi et al. [17]	Increased hospital LOS	Cancer/malignancy	+3.08 days (95% CI: 0.78–5.37), *p* = 0.009
Alqarni et al. [5]	ICU admission	Obesity (BMI ≥ 30)	Adjusted OR 3.34 (95% CI: 1.35–8.22)
Al Sibani et al. [19]	Recurrent exacerbation/readmission risk	Eosinophilic AECOPD phenotype (>0.3 × 10^9^ cells/L)	Median annual readmissions: 1 (IQR 0–3) vs. 0 (IQR 0–1), *p* = 0.04
Alsayed et al. [20]	Acute exacerbation trigger	Human rhinovirus (HRV) positivity	19% (11/58) positivity during exacerbation
Alshehri and Alshibani [10]	ED utilization	* Poor medication adherence	57.5% vs. 44.6% (*p* = 0.036)
Alshehri and Alshibani [10]	Post-ED hospitalization	* Poor medication adherence	33.1% vs. 18.7% (*p* = 0.007)
Alshehri et al. [21]	Increased hospital readmission	Medication non-adherence (reported as clinical variable from patient records)	Not reported
Bener et al. [22]	Increased hospital admission	Air pollution	Not reported
Kokturk et al. [9]	Low medication adherence * Self-reported (Morisky Medication Adherence Scale-8)	Living in Saudi Arabia versus Turkey	OR 3.20 (*p* = 0.0001)
Onadeko et al. [7]	Acute exacerbation management	Acidemia (pH < 7.30)	OR 0.03 (*p* < 0.0001)
Polatli et al. [8]	Lower hospitalization risk	Male gender	OR 0.62 (95% CI: 0.46–0.93)

Predictors and factors associated with acute-care utilization, medication adherence, and clinical outcomes among patients. The reported values are presented as percentages, means ± standard deviations (SD), medians with interquartile ranges (IQR), odds ratios (OR), relative risks (RR), area under the receiver operating characteristic curve (AUROC), or effect estimates with 95% confidence intervals (CIs), as reported in the original studies. * Medication adherence status reported from patient medication-use records/questionnaire methodology.

## Data Availability

No new data were created or analyzed in this study.

## Supplementary Materials

The following supporting information can be downloaded at: https://www.mdpi.com/article/10.3390/healthcare14131962/s1.

## References

[1] Global Initiative for Chronic Obstructive Lung Disease (GOLD) (2026). Global Strategy for the Diagnosis, Management, and Prevention of Chronic Obstructive Pulmonary Disease: 2026 Report.

[2] GBD 2021 Chronic Respiratory Disease Collaborators (2025). Global, regional, and national burden of chronic respiratory diseases and risk factors, 1990–2021: A systematic analysis for the Global Burden of Disease Study 2021. Lancet Respir. Med..

[3] World Health Organization (2024). Chronic Obstructive Pulmonary Disease (COPD) Fact Sheet; Updated 6 November 2024.

[4] Alaithan A.M., Memon J.I., Rehmani R.S., Qureshi A.A., Salam A. (2012). Chronic obstructive pulmonary disease: Hospital and intensive care unit outcomes in the Kingdom of Saudi Arabia. Int. J. Chronic Obstr. Pulm. Dis..

[5] Alqarni A.A., Badr O.I., Aldhahir A.M., Alqahtani J.S., Siraj R.A., Naser A.Y., Alghamdi A.S., Majrshi M., Alghamdi S.M., Alyami M.M. (2024). Obesity prevalence and association with spirometry profiles, ICU admission, and comorbidities among patients with COPD: Retrospective study in two tertiary centres in Saudi Arabia. Int. J. Chronic Obstr. Pulm. Dis..

[6] Vestbo J., Hurd S.S., Agustí A.G., Jones P.W., Vogelmeier C., Anzueto A., Barnes P.J., Fabbri L.M., Martinez F.J., Nishimura M. (2013). Global strategy for the diagnosis, management, and prevention of chronic obstructive pulmonary disease: GOLD executive summary. Am. J. Respir. Crit. Care Med..

[7] Onadeko B.O., Khadadah M., Abdella N., Mukhtar M., Mourou M., Qurtom M., Samad M., Al-Shayeb A. (2005). Prognostic factors in the management of exacerbation of chronic obstructive pulmonary disease in Kuwait. Med. Princ. Pract. Int. J. Kuwait Univ. Health Sci. Cent..

[8] Polatli M., Kheder A.B., Wali S., Javed A., Khattab A., Mahboub B., Iraqi G., Nejjari C., Taright S., Koniski M.-L. (2012). Chronic obstructive pulmonary disease and associated healthcare resource consumption in the Middle East and North Africa: The BREATHE study. Respir. Med..

[9] Kokturk N., Polatli M., Oguzulgen I.K., Saleemi S., Al Ghobain M., Khan J., Doble A., Tariq L., Aziz F., El Hasnaoui A. (2018). Adherence to COPD treatment in Turkey and Saudi Arabia: Results of the ADCARE study. Int. J. Chronic Obstr. Pulm. Dis..

[10] Alshehri S., Alshibani M. (2020). Impact of medication adherence on emergency department visits in patients with COPD in a single tertiary hospital in Saudi Arabia. Int. J. Chronic Obstr. Pulm. Dis..

[11] Saja M.F., Younis A.S., Alzahrani L.A., Alkhlassi I.N., Aldakhil L.S., Albayyat R.M., Alnasser N.A., Alokla K.S., Alghamdi L.A., Rashed A.N. (2025). The Association Between Medication Adherence and Health-Related Quality of Life in Patients with COPD: A Cross-Sectional Study. Int. J. Chronic Obstr. Pulm. Dis..

[12] Tricco A.C., Lillie E., Zarin W., O’Brien K.K., Colquhoun H., Levac D., Moher D., Peters M.D.J., Horsley T., Weeks L. (2018). PRISMA extension for scoping reviews (PRISMA-ScR): Checklist and explanation. Ann. Intern. Med..

[13] Ex Libris (2026). *RefWorks*, [Reference Management Software]. https://refworks.proquest.com.

[14] Aromataris E., Munn Z. (2020). JBI Manual for Evidence Synthesis.

[15] Al-Jahdali H., Ahmed A., Al-Harbi A., Khan A., Algamedi T. (2023). The most common pulmonary diseases length of stay, and characteristics of patients admitted to pulmonary service. Ann. Thorac. Med..

[16] Almarshoodi K., Echevarria C., Kassem A., Mahboub B., Salameh L., Ward C. (2024). An international validation of the “DECAF score” to predict disease severity and hospital mortality in acute exacerbation of COPD in the UAE. Hosp. Pharm..

[17] Alotaibi T.F., Othman F., Alrasheed B.A., Aldraiwish B.M., Alharthi M.M., Alotaibi H.G., Alghamdi A.S., Aljohani H., Alqahtani M.M., Ismaeil T.T. (2025). Length of stay and comorbidity prevalence among individuals with chronic obstructive pulmonary disease in a tertiary care healthcare center in Saudi Arabia; A cross-sectional study. Medicine.

[18] Alqarni A.A., Aldhahir A.M., Ghilani R., Alharbi J., Alharbi T., Alaydarous F., Siraj R., Alqahtani J.S., Alwafi H. (2025). Associations of Psychological and Respiratory Symptoms With Clinical Outcomes Among Patient with Chronic Obstructive Pulmonary Disease. Am. J. Respir. Crit. Care Med..

[19] Al Sibani M., Al Alawi A., Al Aghbari J. (2022). Elevated Peripheral Blood Eosinophils during Acute Exacerbation of Chronic Obstructive Pulmonary Disease: Prevalence and clinical significance. Sultan Qaboos Univ. Med. J..

[20] Alsayed A.R., Abed A., Khader H.A., Hasoun L., Al Maqbali M., Al Shawabkeh M.J. (2024). The role of human rhinovirus in COPD exacerbations in Abu Dhabi: Molecular epidemiology and clinical significance. Libyan J. Med..

[21] Alshehri F., Alghamdi M., Aloqabi F.A., Ibrahim A., Tayeb N., Hassosah M., Abu-Zaid A., Fan H., Vali Y. (2025). Prevalence and Clinical Outcomes of Eosinophilic COPD in a Saudi Population: A Retrospective Study. Saudi J. Med. Med. Sci..

[22] Bener A., Dogan M., Ehlayel M.S., Shanks N.J., Sabbah A. (2009). The impact of air pollution on hospital admission for respiratory and cardiovascular diseases in an oil and gas-rich country. Eur. Ann. Allergy Clin. Immunol..

[23] Alqahtani J.S., Njoku C.M., Bereznicki B., Wimmer B.C., Peterson G.M., Kinsman L., Aldabayan Y.S., Alrajeh A.M., Aldhahir A.M., Mandal S. (2020). Risk factors for all-cause hospital readmission following exacerbation of COPD: A systematic review and meta-analysis. Eur. Respir. Rev..

