# Supplementary material for: Chronic Obstructive Pulmonary Disease in the Gulf Cooperation Council: Acute-Care Utilization and Medication Adherence: A PRISMA-ScR Scoping Review
Source: Healthcare (Basel). 2026 Jul 2;14(13):1962. doi: 10.3390/healthcare14131962 (PMC13362153; doi:10.3390/healthcare14131962)
Supplement: Supplementary file 1 [file healthcare-14-01962-s001.zip › healthcare-4305715-supplementary.pdf]

Supplementary File 1. Search Strategies

Databased searches were performed from inception to 22 May 2026. The following search strategies were used. Limits included English language.

S1. PubMed (MEDLINE)

("Pulmonary Disease, Chronic Obstructive"[MeSH] OR COPD[Title/Abstract] OR "chronic obstructive pulmonary disease"[Title/Abstract] OR AECOPD[Title/Abstract] OR exacerbation*[Title/Abstract]) AND ("Saudi Arabia"[Title/Abstract] OR "United Arab Emirates"[Title/Abstract] OR UAE[Title/Abstract] OR Oman[Title/Abstract] OR Kuwait[Title/Abstract] OR Qatar[Title/Abstract] OR Bahrain[Title/Abstract]) AND (("Hospitalization"[MeSH] OR "Patient Readmission"[MeSH] OR "Emergency Service, Hospital"[MeSH] OR admission*[Title/Abstract] OR readmission*[Title/Abstract] OR rehospital*[Title/Abstract] OR "emergency department"[Title/Abstract] OR hospitalization[Title/Abstract] OR hospitalisation[Title/Abstract]) OR ("Medication Adherence"[MeSH] OR adherence[Title/Abstract] OR complianc*[Title/Abstract] OR nonadheren*[Title/Abstract] OR noncomplianc*[Title/Abstract] OR persist*[Title/Abstract] OR "medication possession ratio"[Title/Abstract] OR MPR[Title/Abstract] OR PDC[Title/Abstract] OR "proportion of days covered"[Title/Abstract] OR Morisky[Title/Abstract] OR MMAS[Title/Abstract] OR "MMAS-8"[Title/Abstract] OR "Morisky Medication Adherence Scale"[Title/Abstract] OR "inhaler adherence"[Title/Abstract] OR "inhaler compliance"[Title/Abstract]))

S2. Google Scholar:

(“COPD” OR “chronic obstructive pulmonary disease”)
AND
(“Saudi Arabia” OR “United Arab Emirates” OR UAE OR Oman OR Kuwait OR Qatar OR Bahrain)
AND
((“acute exacerbation” OR hospitalization OR readmission OR “emergency department”)
OR
(“medication adherence” OR compliance))

S3. Embase:

('chronic obstructive lung disease'/exp OR copd:ti,ab OR 'chronic obstructive pulmonary disease':ti,ab OR 'chronic obstructive lung disease':ti,ab OR 'obstructive lung disease':ti,ab) AND ('saudi arabia':ti,ab OR 'united arab emirates':ti,ab OR uae:ti,ab OR oman:ti,ab OR kuwait:ti,ab OR qatar:ti,ab OR bahrain:ti,ab) AND ('acute exacerbation':ti,ab OR exacerbation*:ti,ab OR hospitalization:ti,ab OR readmission*:ti,ab OR 'emergency department':ti,ab OR 'acute care':ti,ab OR 'medication adherence':ti,ab OR adherence:ti,ab OR compliance:ti,ab OR 'medication possession ratio':ti,ab OR morisky:ti,ab OR 'inhaler adherence':ti,ab OR 'proportion of days covered':ti,ab)

S4. Scopus:

TITLE-ABS-KEY ((COPD OR "chronic obstructive pulmonary disease" OR "chronic obstructive lung disease" OR "obstructive lung disease") AND ("Saudi Arabia" OR "United Arab Emirates" OR UAE OR Oman OR Kuwait OR Qatar OR Bahrain) AND (("acute exacerbation" OR exacerbation* OR hospitalization OR readmission* OR "emergency department" OR "acute care") OR ("medication adherence" OR adherence OR compliance OR "medication possession ratio" OR Morisky OR "inhaler adherence" OR "proportion of days covered")))

S5. Cochrane Library:

(COPD OR "chronic obstructive pulmonary disease" OR "chronic obstructive lung disease" OR "obstructive lung disease") AND ("Saudi Arabia" OR "United Arab Emirates" OR UAE OR Oman OR Kuwait OR Qatar OR Bahrain) AND (("acute exacerbation" OR exacerbation* OR hospitalization OR readmission* OR "emergency department" OR "acute care") OR ("medication adherence" OR adherence OR compliance OR "medication possession ratio" OR Morisky OR "inhaler adherence" OR "proportion of days covered")) in Title Abstract Keyword - (Word variations have been searched)

Supplementary Materials

Supplementary A: Tables

Supplementary Table A. Preferred Reporting Items for Systematic Reviews and Meta-Analyses extension for Scoping Reviews (PRISMA-ScR) Checklist

SECTION	ITEM	PRISMA-ScR CHECKLIST ITEM	REPORTED ON PAGE #
**TITLE**			
Title	1	Identify the report as a scoping review.	Page 1
**ABSTRACT**			
Structured summary	2	Provide a structured summary that includes (as applicable): background, objectives, eligibility criteria, sources of evidence, charting methods, results, and conclusions that relate to the review questions and objectives.	Page 1
**INTRODUCTION**			
Rationale	3	Describe the rationale for the review in the context of what is already known. Explain why the review questions/objectives lend themselves to a scoping review approach.	Page 2
Objectives	4	Provide an explicit statement of the questions and objectives being addressed with reference to their key elements (e.g., population or participants, concepts, and context) or other relevant key elements used to conceptualize the review questions and/or objectives.	Page 2-3
**METHODS**			
Protocol and registration	5	Indicate whether a review protocol exists; state if and where it can be accessed (e.g., a Web address); and if available, provide registration information, including the registration number.	Page 3
Eligibility criteria	6	Specify characteristics of the sources of evidence used as eligibility criteria (e.g., years considered, language, and publication status), and provide a rationale.	Page 3
Information sources*	7	Describe all information sources in the search (e.g., databases with dates of coverage and contact with authors to identify additional sources), as well as the date the most recent search was executed.	Page 3 and Appendix

SECTION	ITEM	PRISMA-ScR CHECKLIST ITEM	REPORTED ON PAGE #
Search	8	Present the full electronic search strategy for at least 1 database, including any limits used, such that it could be repeated.	Supplementary File 1
Selection of sources of evidence†	9	State the process for selecting sources of evidence (i.e., screening and eligibility) included in the scoping review.	Supplementary File 1
Data charting process‡	10	Describe the methods of charting data from the included sources of evidence (e.g., calibrated forms or forms that have been tested by the team before their use, and whether data charting was done independently or in duplicate) and any processes for obtaining and confirming data from investigators.	Page 3
Data items	11	List and define all variables for which data were sought and any assumptions and simplifications made.	Page 3 and Supplementary
Critical appraisal of individual sources of evidence§	12	If done, provide a rationale for conducting a critical appraisal of included sources of evidence; describe the methods used and how this information was used in any data synthesis (if appropriate).	Page 4
Synthesis of results	13	Describe the methods of handling and summarizing the data that were charted.	Page 4
**RESULTS**			
Selection of sources of evidence	14	Give numbers of sources of evidence screened, assessed for eligibility, and included in the review, with reasons for exclusions at each stage, ideally using a flow diagram.	Page 4-5
Characteristics of sources of evidence	15	For each source of evidence, present characteristics for which data were charted and provide the citations.	Page 5-9 (Table 1)
Critical appraisal within sources of evidence	16	If done, present data on critical appraisal of included sources of evidence (see item 12).	Page 11-12 (Table 2)
Results of individual sources of evidence	17	For each included source of evidence, present the relevant data that were charted that relate to the review questions and objectives.	Page 5-14
Synthesis of results	18	Summarize and/or present the charting results as they relate to the review questions and objectives.	Page 10-15
**DISCUSSION**			
Summary of evidence	19	Summarize the main results (including an overview of concepts, themes, and types of evidence available), link to the review questions and	Page 15-16

SECTION	ITEM	PRISMA-ScR CHECKLIST ITEM	REPORTED ON PAGE #
		objectives, and consider the relevance to key groups.	
Limitations	20	Discuss the limitations of the scoping review process.	Page 16
Conclusions	21	Provide a general interpretation of the results with respect to the review questions and objectives, as well as potential implications and/or next steps.	Page 16-17
**FUNDING**			
Funding	22	Describe sources of funding for the included sources of evidence, as well as sources of funding for the scoping review. Describe the role of the funders of the scoping review.	Page 17

JBI = Joanna Briggs Institute; PRISMA-ScR = Preferred Reporting Items for Systematic reviews and Meta-Analyses extension for Scoping Reviews.
* Where *sources of evidence* (see second footnote) are compiled from, such as bibliographic databases, social media platforms, and Web sites.
† A more inclusive/heterogeneous term used to account for the different types of evidence or data sources (e.g., quantitative and/or qualitative research, expert opinion, and policy documents) that may be eligible in a scoping review as opposed to only studies. This is not to be confused with *information sources* (see first footnote).
‡ The frameworks by Arksey and O'Malley (6) and Levac and colleagues (7) and the JBI guidance (4, 5) refer to the process of data extraction in a scoping review as data charting.
§ The process of systematically examining research evidence to assess its validity, results, and relevance before using it to inform a decision. This term is used for items 12 and 19 instead of "risk of bias" (which is more applicable to systematic reviews of interventions) to include and acknowledge the various sources of evidence that may be used in a scoping review (e.g., quantitative and/or qualitative research, expert opinion, and policy document).

From: Tricco AC, Lillie E, Zarin W, O'Brien KK, Colquhoun H, Levac D, et al. PRISMA Extension for Scoping Reviews (PRISMAScR): Checklist and Explanation. Ann Intern Med. 2018;169:467–473. doi: 10.7326/M18-0850.

Supplementary Table. Inclusion and exclusion criteria for source selection

Criteria	Inclusion	Exclusion
**Research focus**	COPD related acute-care utilization and/or medication adherence; GCC-country-specific data (Bahrain, Kuwait, Oman, Qatar, Saudi Arabia, the UAE); human participants	Not COPD population; no acute-care utilization or adherence outcome; no GCC-specific results (region aggregated only); non-human/in vitro
**Study design**	Observational and interventional quantitative studies	Studies were excluded if they were not COPD-focused, lacked extractable GCC-specific data, included mixed respiratory populations without separable COPD-specific outcomes, focused exclusively on physiologic outcomes without utilization or adherence endpoints, or represented reviews, editorials, commentaries, protocols, conference abstracts without extractable data, or other non-primary research publications
**Language**	English	Non-English
**Publication year**	Database inception to the last search date	Published after the last search date
**Population**	Adults with clinician- or spirometry-confirmed COPD; severe exacerbation cohorts; COPD-coded administrative datasets when the COPD case definition is stated	Asthma-only; non-COPD respiratory conditions without separable COPD subgroup; pediatric-only populations; mixed cohorts without extractable COPD-specific results
**Setting and geography**	GCC countries; any care setting (ED, inpatient, ICU, outpatient, community)	Outside GCC; multi-country studies with only aggregated results (no GCC-country estimates)
**Outcomes (≥1 required)**	COPD related acute-care utilization outcomes included COPD hospitalization, intensive care unit (ICU) admission, all-cause emergency department (ED) visits, mortality, and readmission. Medication-adherence outcomes included adherence or treatment-utilization measures.	No target outcomes; COPD prevalence-only studies; physiologic-only studies without utilization/adherence endpoints
**Publication type/status**	Peer-reviewed full-text articles;	Abstract-only records without extractable data; non-scholarly media; commentary pieces

**Full-text availability**	Full text retrievable (open access, institutional access, author contact) with sufficient detail for screening and data charting	Full text not available
